# *ALS2*-related disorders in Spanish children

**DOI:** 10.1007/s10072-020-04899-0

**Published:** 2021-01-07

**Authors:** Enrique Nogueira, Juana Alarcón, Carmen Garma, Cecilia Paredes

**Affiliations:** 1 Molecular Diagnostics Eurofins-Megalab, Hospital San Rafael, Madrid, Spain; 2 Genetics Service, Hospital La Zarzuela, Madrid, Spain; 3 Pediatric Neurology, Hospital San Rafael, Madrid, Spain

**Keywords:** *ALS2*, *ALS2*-related disorders, *Infantile ascending hereditary spastic paraplegia*, IAHSP, Spanish children

## Abstract

*ALS2 *gene encoding for alsin protein is responsible for neurological disorders due to retrograde degeneration of the upper motor neurons of the pyramidal tracts, inherited in an autosomal recessive manner, and displaying a clinical continuum including the infantile ascending hereditary spastic paraplegiaidentified in three Spanish children presented here.

Dear Editor-in-Chief,

We hereby report on three ALS-like cases in Spanish children linked to gene *ALS2* (*alsin Rho guanine nucleotide exchange factor ALS2*) of 2q33.1 encoding alsin protein. *ALS2*-related disorders are considered a consequence of retrograde degeneration of upper motor neurons (UMN) of pyramidal tracts displaying a clinical *continuum* with infantile to juvenile onset and a recessive inheritance pattern, ranging from IAHSP (*infantile ascending hereditary spastic paraplegia*) and JPLS (*juvenile primary lateral sclerosis*), both primarily assigned to UMN, to JALS (*juvenile amyotrophic lateral sclerosis*), with additional lower motor neuron (LMN) involvement [1, 2].

The prevalence of *ALS2* disorders is unknown, with only a few cases having been described in a variety of ethnic backgrounds including Caucasians. Among the latter, the first Spanish patients reported hereby were three children of non-consanguineous parents, from *Castilla La Mancha* (two sibs in family 1, F1) and the *Basque Country* (case in family 2, F2). *ALS2* mutations were initially discovered in F1 *index case* and the F2 case submitted to a genome analysis for *spastic paraplegia* undertaken by *next generation sequencing* (NGS) with the use of the *TruSight One Sequencing Panel* (https://www.illumina.com), leading to identification of two *compound heterozygous* genotypes, that clearly underneath disease, of *ALS2* mutations Q982SfsX19 and R704X in F1 and R640X and G49R in F2. They are presented in Figs. 1 and 2 with the available clinical data of the patients as well as in Fig. 3 to show its location in the alsin sequence together with many of the *ALS2* mutations described from 2001 to 2018 [3–20].

**Fig. 1:**
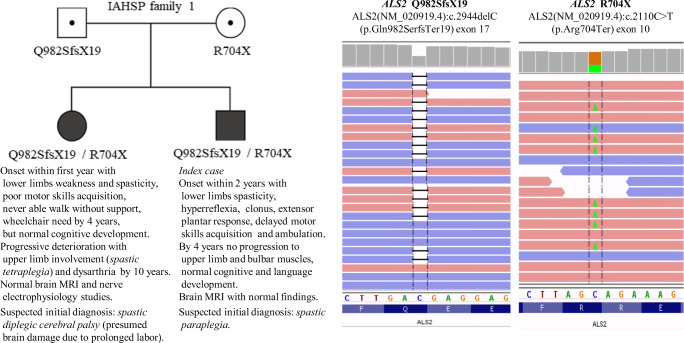
Relevant features of IAHSP family 1(F1) patients together with illustrations of ALS2 mutations

**Fig. 2:**
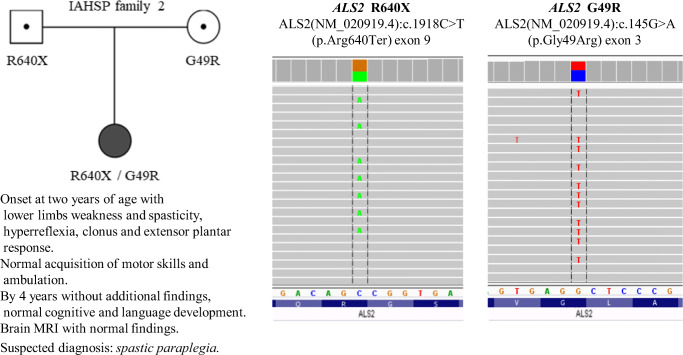
Relevant features of IAHSP family 2 (F2) patient together with illustrations of ALS2 mutations

**Fig. 3:**
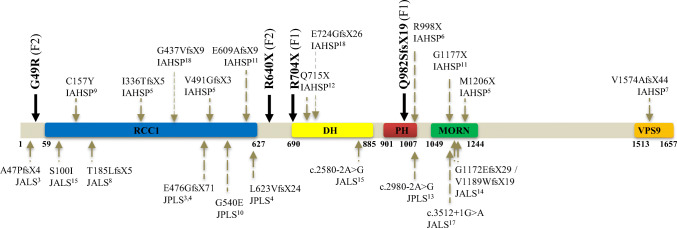
Disposition of the *ALS2* mutations identified in the Spanish children (in bold) and some other patients throughout the alsin sequence. Mutations G49R and R640X had previously been described in AIHSP patients^16,19^. IAHSP, *infantile ascending hereditary spastic paraplegia*; JALS, *juvenile amyotrophic lateral sclerosis*; JPLS, *juvenile primary lateral sclerosis*; RCC1, *regulator of chromatin condensation 1*; DH/PH, *Dbl and Pleckstrin homology*; MORN: *membrane occupation and recognition nexus*; VPS9, *vacuolar protein sorting 9*

F1 mutations Q982SfsX19 and R704X have been identified for the first time as far as we know. However, they are considered *pathogenic* taking into account their deleterious nature, location, and familial segregation. They are indeed frameshift (Q982SfsX19, paternal derived) and nonsense (R704X, maternal) variants like most of pathogenic *ALS2* mutations that predict truncated defective proteins and are located in DH/PH domain near to pathogenic variants of similar type such as E724GfsX26 and Q715X. Of these, E724GfsX26 was described with variant G437VfsX9 of RCC1 domain in a *compound heterozygous* genotype in two Chinese siblings with IAHSP [18], whereas the homozygous nonsense mutation Q715X was reported in two sibs claimed to be the first IAHSP cases from northwestern Europe [12]. Besides, the pathogenic nature of *ALS2* F1 mutations is pointed out by its segregation. Each mutation is clinically silent in the parents but together in a *compound heterozygous* genotype is associated to disease in the siblings though with remarkable differences. Whereas *index case* would be an apparent typical IAHSP case, that of his old sister much more severe might suggest the consideration of an early-onset JPLS. A similar of severe picture has recently been observed in a young girl of Seville (Spain) homozygous for the R704X mutation (Nogueira E, unpublished).

F2 patient would be a typical IAHSP case due to a *compound heterozygous ALS2* genotype of two previously reported mutations, R640X, of paternal derivation, and G49R, maternal. The nonsense R640X mutation that lies in a sequence between RCC1 and DH domains has been found in homozygosis in two Pakistani siblings considered IAHSP cases [19]. On the other hand, the missense mutation G49R located upstream of RCC1 domain has also been described as part of a *compound heterozygous ALS2* genotype, together with mutation G477AfsX19 in a boy described as first Portuguese IAHSP case [16]. In addition to its segregation in the Portuguese and our F2 cases, the relevant nature of G49R is suggested by its very rare occurrence (only twice in *genomAD* databases, with 0.0004% global allelic frequency) and by affecting a very conserved residue (Gly^49^) whose *non-conservative* substitution (by Arg) would probably have conformational and functional consequences, a presumption congruent with the pathogenic prediction of a majority of in silico analyses as described in knowledge base *VarSome* (https://varsome.com). Thus, according to the ACMG evaluation criteria, G49R might be considered a *pathogenic* or *likely pathogenic* variant. It would increase the small number of missense variants described in different *ALS2* disorders, such as S100I, C157Y, and G540E with proposed association to cases of JALS, IAHSP, and JPLS, respectively (Fig. 3). According to recent data, the pathogenic nature of missense mutations would be due to altered oligomerization and/or destabilization of the mutant alsin molecules impairing its endosomal localization [21].

The finding of *ALS2* mutations in our F1 and F2 families allowed a precise diagnosis of the patients despite of the scarce clinical data suggesting cases of *spastic paraplegia*. They would be *ALS2*-related disorders of the *UMN spectrum*, most probably of IAHSP type although considering the marked affectation differences of F1 siblings, and it is tempting to speculate that same genotype (Q982SfsX19/R704X) might be associated to different disorders, being that of *index case* a typical example of IAHSP whereas that of his old sister would also be compatible with diagnosis of an early-onset JPLS. Such an observation is in line with the known intra- and interfamilial phenotypic variability of *ALS2*-related disorders, noted among others in 11 IAHSP patients of three unrelated consanguineous Iranian families homozygous for mutation c.1640+1G>A of *ALS2* [20]. Of them, three siblings from one family exhibited dystonia not been in families with IAHSP, only previously described in unrelated consanguineous families with JALS/ALS2 [16, 20]. A marked phenotypic variability has also been observed in two siblings homozygous for *ALS2* variant c.2980-2A>G, considered JPL cases, one of them began using a wheelchair at the age of two whereas the other began using it at age 50 [13].

Further, the distribution of F1 and F2 mutations as well as other *ALS2* mutations (illustrated by Fig. 3) is in favor of a lack of both mutation hotspots and a precise domain involvement in the different *ALS2*-related disorders that otherwise due to considerable clinical overlap led to divergent diagnosis, in particular of *UMN spectrum* disorders being in some instances similar pictures called either IAHSP or JPLS [1]. These observations and the phenotypic variability of same genotypes suggest the consideration of a decisive contribution of additional factors in the pathogenesis of *ALS2* disorders, mainly of genes coding for molecules interacting with alsin along its multiple functions, particularly in the endolysosomal pathway [21], that may display functional variations due even to subtle changes, no necessarily to pathogenic variants, attributable to diversity of the genomic dotation of *ALS2* mutation carriers including of siblings.
